# Whole Blood Transcriptome Analysis of *Mycoplasma mycoides* Subsp. *mycoides*-Infected Cattle Confirms Immunosuppression but Does Not Reflect Local Inflammation

**DOI:** 10.1371/journal.pone.0139678

**Published:** 2015-10-02

**Authors:** Valérie Rodrigues, Philippe Holzmuller, Carinne Puech, Hezron Wesonga, François Thiaucourt, Lucía Manso-Silván

**Affiliations:** 1 CIRAD, UMR15 CMAEE, F-34398 Montpellier, France; 2 INRA, UMR1309 CMAEE, F-34398 Montpellier, France; 3 Kenyan Agricultural Research Institute, Nairobi, Kenya; Auburn University, UNITED STATES

## Abstract

Contagious bovine pleuropneumonia (CBPP), caused by *Mycoplasma mycoides* subsp. *mycoides (Mmm)*, is a severe respiratory disease of cattle responsible for major economic losses in sub-Saharan Africa. Disease control relies mainly on the use of empirically attenuated vaccines that provide limited protection. Thus, understanding the virulence mechanisms used by *Mmm* as well as the role of the host immune system in disease development, persistence, and control is a prerequisite for the development of new, rationally designed control strategies. The aim of this study was to assess the use of whole blood transcriptome analysis to study cattle-*Mmm* interactions, starting by the characterization of the bovine response to *Mmm* infection during the acute form of the disease. For that purpose, we compared the transcriptome profile of whole blood from six cattle, before challenge by contact with *Mmm*-infected animals and at the appearance of first clinical signs, using a bovine microarray. Functional analysis revealed that 680 annotated genes were differentially expressed, with an overwhelming majority of down-regulated genes characterizing an immunosuppression. The main bio-functions affected were “*organismal survival”*, *“cellular development*, *morphology and functions”* and *“cell-to cell signaling and interactions”*. These affected functions were consistent with the results of previous *in vitro* immunological studies. However, microarray and qPCR validation results did not highlight pro-inflammatory molecules (such as TNFα, TLR2, IL-12B and IL-6), whereas inflammation is one of the most characteristic traits of acute CBPP. This global gene expression pattern may be considered as the result, in blood, of the local pulmonary response and the systemic events occurring during acute CBPP. Nevertheless, to understand the immune events occurring during disease, detailed analyses on the different immune cell subpopulations, either *in vivo*, at the local site, or *in vitro*, will be required. Whole blood transcriptome analysis remains an interesting approach for the identification of bio-signatures correlating to recovery and protection, which should facilitate the evaluation and validation of novel vaccine formulations.

## Introduction

Contagious bovine pleuropneumonia (CBPP) is an inflammatory respiratory disease caused by *Mycoplasma mycoides* subsp. *mycoides*, previously known as “Small Colony” (*Mmm*) [1]. CBPP is a severe disease of cattle and is responsible for substantial economic losses in sub-Saharan Africa. Given its impact on livestock production and its potential for spread, CBPP is on the list of notifiable diseases of the World Organization for Animal Health (OIE) [2].

Bovids and water buffaloes are the only natural hosts of concern here, and the disease is only transmitted by direct contact with infected animals. *Mmm* shows a strict tropism to the thoracic cavity, where it induces a severe inflammatory reaction characterized by serofibrinous pleurisy and interstitial pneumonia, whereby lesions typically affect only one lung [3]. This classical disease presentation is observed in the acute form of the disease, when *Mmm* infects naïve populations, and results in a high mortality rate. However, some animals may gradually recover, acting as asymptomatic carriers and excreting the etiologic agent for many months. The sub-acute and chronic forms are less conspicuous and are characterized by the presence of pleural adhesions and encapsulated necrotic lesions, known as “sequestra”.

CBPP is enzootic in African countries, and control relies mainly on vaccination. Unfortunately, the current vaccine strain T1/44 is not optimally efficient, since it confers only partial, transient protection and may provoke side effects due to residual virulence [4]. The strain has been attenuated empirically, and the molecular basis of attenuation is unknown. In this context, understanding the mechanisms behind *Mmm* virulence as well as the role of the host immune system in disease development, persistence, and control is a prerequisite for the development of novel, rationally designed vaccines.

So far, no classical virulence factors such as adhesins or toxins have been identified in the *Mmm* genome [5] and virulence has been attributed to surface or secreted components and intrinsic metabolic functions. In particular, the release of hydrogen peroxide through glycerol metabolism resulting in cellular damage and inflammation, has been proposed as a major virulence factor [6], while capsular and secreted exopolysaccharide (galactan) have been suggested to be involved in disease exacerbation and persistence by enabling resistance to host defenses [7]. *In vitro* immunological studies showed that viable *Mmm* is able to induce programed cell death in bovine blood leukocytes [8] and to depress bovine cell responsiveness to the mitogen Concanavalin A [9], suggesting that *Mmm* has developed mechanisms to prevent its direct elimination by the host immune system.

*Mmm*-specific IFNγ producing memory CD4^+^ T cells have been correlated with disease recovery, suggesting a role for this response in protection [10,11,12,13,14], though Sacchini *et al* reported only a minor role for CD4^+^ lymphocytes in the control of primary *Mmm* infection [15]. A better assessment of the protective immune mechanisms that prevent clinical disease may be obtained through a comprehensive, dynamic characterization of the host response during acute and chronic disease, recovery and protection, using high-throughput genome-wide transcriptomics. Blood is easy to obtain from animals, without the need for slaughter, and previous studies have shown that immune responses detected in blood reflect those at the site of infection [16]. The aim of this study was thus to assess the use of whole blood transcriptome analysis to study host-pathogen interactions in CBPP. In this first study, this type of analysis was used to characterize the bovine response to *Mmm* infection in the acute form of the disease.

The analysis of global gene expression in blood was shown to be an interesting, non-invasive approach to study bovine-mycoplasma interactions. This preliminary analysis provided a global picture of the circulating response in blood during acute CBPP but it did not reflect the local inflammatory disease. Still, this approach may be applied to the identification of some of the molecular mechanisms correlating to disease exacerbation or to recovery and protection (bio-marker signatures), thus paving the way for the development of novel vaccine formulations.

## Material and Methods

### Ethic statement

Scacchia *et al*. showed that only naturally infected cattle are relevant for immune studies on CBPP, as opposed to animals infected by endotracheal intubation [17]. Studies on cattle must therefore be performed under controlled conditions using animals infected by direct contact. In the present study, the experiment was carried out with the permission of the VRC Muguga Institutional Animal Care and Use Committee (IACUC), reference number KARI/VRC/IACUC/2/00122010. Animals were euthanized if they showed fever for 10 days or were recumbent for 3 days. The rest of the animals, without these severe clinical signs, were killed at the end of the experiment. Animals were sacrificed by stunning with a captive bolt, followed by exsanguination, in accordance with the ethical considerations of the VRC Muguga IACUC; analgesics or anesthetics were not used.

### Experimental design and sampling

The status of naïve zebus above three years of age was checked for the absence of *Mmm*-specific antibodies using the cELISA “*Mmm* antibody test kit” (IDEXX, Montpellier, France), a method for CBPP serodiagnosis prescribed by the OIE. To guarantee the natural mode of transmission, 10 naïve animals were put in contact with 20 cattle previously infected with *Mmm* (strain B237) by the endobronchial route. Clinical signs (i.e. fever and cough, at rest or following effort) were checked daily. Following ethical principles, animals that developed severe clinical signs were humanely slaughtered before the end of the experiment.

Sera were collected at regular intervals from the beginning of the trial until slaughter. *Mmm*-specific antibody responses were assessed using the CBPP cELISA twice before contact (10 and 2 weeks before contact), and then monthly after contact until the day of slaughter, using the CBPP cELISA according to the manufacturer’s instructions.

For transcriptome analyses, the first blood sample was collected from all animals 10 weeks before the introduction of infected cattle. Thereafter, blood was sampled from six cattle at the appearance of CBPP clinical signs, which took place 10 to 11 weeks after contact. Tempus^TM^ blood RNA collecting tubes (Applied Biosystems Ltd., Warrington, UK) were used according to manufacturer’s instructions. Briefly, 3 ml of blood was collected from the jugular vein into a Tempus^TM^ tube, and then stored in Kenya at -20°C until shipment at the end of the experimental trial. Samples were transported in refrigerated boxes and stored at -80°C for several weeks until processing using Tempus^TM^ technology and quality control tools.

Necropsy was performed on all animals at slaughter. The presence, type, and size of the pulmonary lesions were recorded.

### RNA extraction and preparation and microarray hybridization

All Tempus samples were treated separately, at the same time. Total RNA was extracted using the Tempus^TM^ Spin RNA Isolation Reagent Kit (Applied Biosystems Ltd., Warrington, UK) according to the manufacturer’s instructions, including optional DNAse treatment. RNA samples were stored at -80°C in multiple aliquots to avoid repeated freeze-thaw cycles. An aliquot of each RNA sample was used to assess RNA yield and quality, using a Nanodrop™ ND-1000 Spectrophotometer (Thermo Fisher Scientific, MA, USA). RNA quality was further assessed using the Agilent 2100 Bioanalyzer platform (Agilent Technologies, Inc. Santa Clara, USA). All the RNA samples were satisfactory, both in terms of quantity and quality, for transcriptome analysis using micro-array technology. We collected 14.8 ± 2.5 μg RNA, with an RNA integrity number (RIN) of 6.9 ± 0.4 from non-infected animals and 7.4 ± 2.6 μg RNA with a RIN of 8.5 ± 0.1 from infected animals.

To produce Cy3-labeled cRNA, each total RNA sample was amplified and labeled using the Agilent Low Input Quick Amplification Labeling Kit, v6.5, (available online: http://www.chem.agilent.com/library/usermanuals/) strictly following the manufacturer’s instructions. All samples were processed in the same manner within a week interval (Four animals first then the remaining two). Briefly, 50 ng of template RNAs were used for cDNA synthesis. T7 RNA polymerase was then used for cRNA amplification and Cy3 labeling. After purification, yield and dye incorporation were measured using a Nanodrop spectrophotometer. A total of 1.65 μg of at least 6 pmol Cy3/ng of each RNA sample was fragmented using the Agilent Gene Expression Hybridization Kit. Sample hybridizations were performed on an Agilent Bovine (V2) Gene Expression Microarray for 17 h at 65°C. After gentle washing, according to Agilent’s protocol, microarrays were scanned on a C scanner (Agilent). Agilent Feature Extraction software (v10.7) was used to read out and process the microarray image files. The software determined feature intensities and background, rejected outliers, and generated validated data files for further analyses. Validation of each array consisted in the analysis of additive error, spatial distribution of outliers on the array, histogram of signal plots, background sub-signal value and linear range spike-in statistics. All assays were satisfactory for these parameters.

### Microarray data normalization and analysis

All validated scan data files were processed using the Agilent GeneSpring GX (v11.5.1) software for normalization with the 75 percentile model, with baseline transformation based on the median of all samples. All entities with flag values present in at least 100% of the values in any one out of the two status conditions (non-infected versus infected) were considered. Statistical analysis (paired t-test) using the volcano plot filter was used to generate a unique list of up- or down-regulated entities with associated Benjamini Hochberg False Discovery Rate corrected p-value and fold change (FC) and for hierarchical clustering.

Using Ingenuity Pathway Analysis (IPA) software, differentially expressed entities were separated into unmapped and mapped entities. A deduplication exercise on mapped entities resulted in a final list of unique identified genes (p-value < 0.05 and absolute fold change ≥ 2), which was then used to perform IPA bio-function analysis. IPA categorized and subcategorized modulated genes according to p-values (calculated by the Fisher exact test) and z-scores. The z-score predicts the direction of change of a function: a function is increased when z-score is ≥ 2 and decreased when z-score ≤ 2. IPA calculated a bias-corrected z-score to correct dataset bias (i.e. when there are more up- than down-regulated genes in a bio-function or vice-versa).

### Quantitative PCR

Six genes were selected for the validation of the microarray results by quantitative reverse trancription-PCR (RT-qPCR) analysis: two down-regulated genes (CD28 and SMAD-5) and four statistically up-regulated genes (IL-1RN, IL-10RA, MyD88, and CXCR2). Four non-modulated genes involved in inflammatory response or pathogen recognition (TLR-2, IL12-B, IL-6 and TNFα) were also tested by RT-qPCR.

In addition, reference genes required for the normalization were determined in all samples as described by Puech [18]. Briefly, the gene expression stability of five reference genes (ACTB, GAPDH, H3F3A, PPIA, and YWHAZ) was determined and calculated using the geNorm analysis [19]. Two reference genes were required (V-value = 0.14) and the two most stable reference genes were GAPDH and ACTB (M-value = 0.844 and 0.883, respectively).

The validation of microarray results was performed using the Cow RT^2^ qPCR assay (Qiagen, Crawley, UK). RT-qPCR was performed with the same RNA samples used for microarray hybridizations. Briefly, 400 nanograms of total RNA from each sample were reverse-transcribed with the RT² First Strand Kit, including genomic DNA elimination step, and according to Qiagen’s instructions. In addition, RNA quality control was checked using Cow RT² RNA QC PCR Array (Qiagen), which proved the efficiency of reverse transcription and the absence of genomic DNA contamination in all samples. QPCR reactions were conducted on Mx3005P QPCR Systems™ (Agilent Technologies, Santa Clara, USA). Amplifications were performed with RT² SYBR Green ROX^TM^ qPCR Mastermix and using RT^2^ qPCR primers (Qiagen). Primer pairs were selected on the same gene sequences (same GenBank accession numbers) as microarray probes (Table 1). The PCR thermal cycling program consisted in one step at 95°C for 10 min, followed by 40 cycles of 95°C for 15 s and 60°C for 1 min, and a final dissociation step, which confirmed single specific PCR product amplifications. A negative control (no-template control) was included in each primer assay.

**Table 1:** qPCR and microarray GeneBank accession numbers and Fold Changes of tested genes.

Gene	GenBank accession number	FC Array	FC REST normalised
*CD28*	NM_181004.1	**-2.72**	**-1.653**
*IL-1RN*	NM_174357.3	**3.058**	1.325
*IL-10RA*	NM_001205757.1	**2.923**	1.094
*MyD88*	NM_001014382.2	**3.202**	1.391
*CXCR2*	NM_001101285.1	**4.06**	1.473
*SMAD5*	NM_001077107.2	**-5.243**	**-1.587**
*TNFα*	NM_173966.3	-	-1.31
*TLR2*	NM_174197.2	-	-1.19
*IL-12B*	NM_174356.1	-	1.386
*IL-6*	NM_173923.2	-	**-2.32**

*Bold = p-value<0*.*05;*

FC of expression levels between infected and control groups were calculated using the relative expression software tool (REST) [20]. Correlation between the microarray and RT-qPCR results for the six regulated genes was performed by the Pearson’s correlation calculation (www.socscistatistics.com/tests/pearson/) and the statistical significance of the correlations was determined. For microarray results, the data input in the correlation analysis was the absolute FC value for each gene (S1 Table). For RT-qPCR, the normalized expression results calculated by REST were used.

## Results

### Serological and post-mortem analyses show efficient CBPP transmission

**Fig 1:**
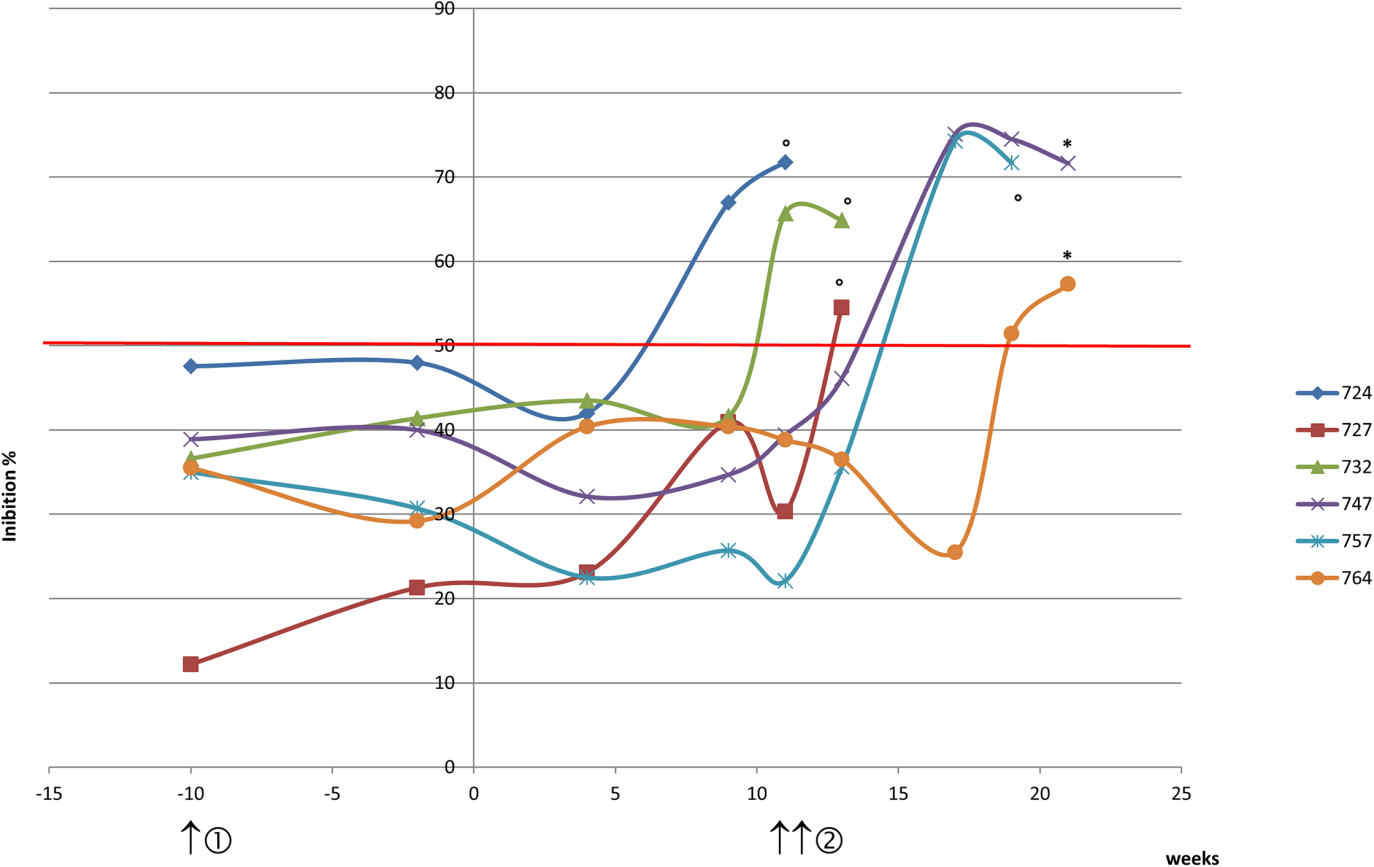
Experimental protocol and cELISA CBPP kinetics. Healthy zebus were put in contact with *Mmm*-infected cattle (time = 0). *Mmm*-specific antibody responses were assessed twice before contact, monthly after contact, and on the day of slaughter, using the CBPP cELISA test (IDEXX, Montpellier, France). The seropositivity threshold, defined by inhibition percentage values > 50%, is represented by the red horizontal line. For transcriptome analyses, blood samples were collected before the introduction of infected cattle (①, time = -10 weeks) and subsequently, blood was sampled from six newly infected zebus when CBPP clinical signs appeared (②, time = 10 weeks for 724, 727 and 732 and 11 weeks for animals 747, 757 and 764). ° Animal euthanized because of severe clinical signs. * Animal slaughtered at the end of the experiment.

Ten naive zebus above three years of age were put in contact with *Mmm*-infected cattle to ensure natural transmission. Clinical signs were checked daily and six cattle showing typical CBPP signs at approximately the same time were chosen for whole blood transcriptome analysis. The experimental protocol is outlined in Fig 1.

According to CBPP cELISA results, all six animals were seropositive after nine to nineteen weeks of contact with *Mmm*-infected animals. Four of them became seropositive after the beginning of the clinical signs, when the second blood sample for transcriptome analysis was collected. The in-contact exposure route, less controlled than the intra-tracheal inoculation for experimental challenge, still allowed us to obtain six animals showing clinical signs at approximately the same time. All animals showed clinical signs after two and a half months of contact with *Mmm*-infected cattle: 10 weeks for animals 724, 727 and 732 and 11 weeks for animals 747, 757 and 764 (Fig 1). Animals 724, 727, 732 and 757 were euthanized before the end of the experiment, due to severe clinical signs, whereas animals 747 and 764 were euthanized at the end of the experiment, after 5 months of contact with *Mmm*-infected cattle. Post-mortem analysis showed that all the animals presented lesions considered characteristic of acute CBPP, with lesions more than 20 cm in size for animals 724, 727, 732 and 764, and smaller lesions for animals 747 (9x12cm) and 757 (12x16cm).

### Cluster analysis shows a clear distinction between expression profiles of *Mmm*-infected and non-infected animals

We investigated gene expression in the whole blood of six cattle before and after contact with *Mmm-*infected cattle, using an Agilent Bovine Gene Expression Microarray, containing 43,654 probes (entities) and representing 35,028 annotated genes. Results of a GeneSpring paired t-test revealed that 1,115 entities (2.5% of probes) were significantly differentially expressed (absolute fold change ≥ 2, with a Benjamini Hochberg False Discovery Rate corrected p-value ≤ 0.05).

A notable feature was a higher proportion of down-regulated (947, i.e. 84.9%) than up-regulated entities (168, i.e. 15.1%).

**Fig 2:**
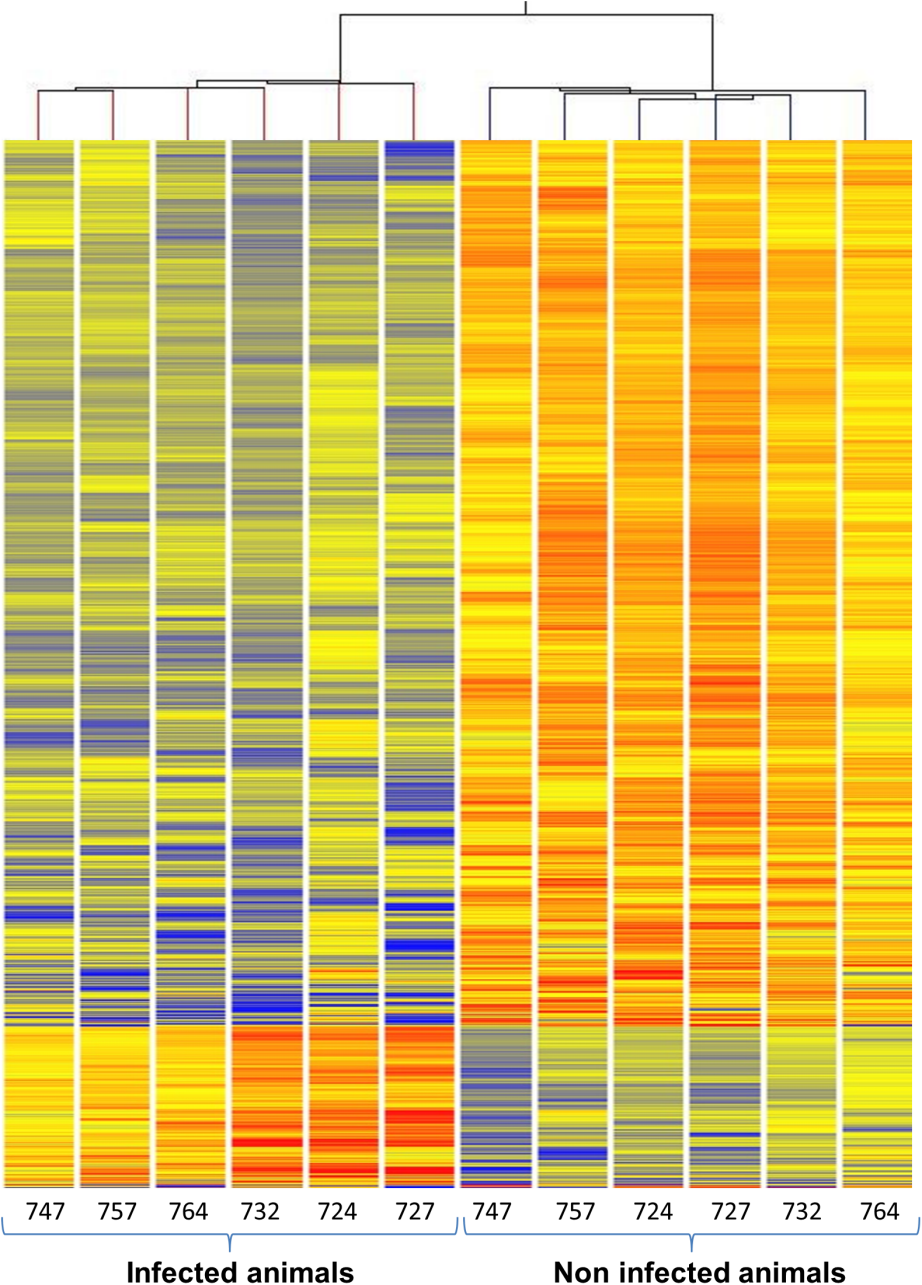
Hierarchical combined tree. Hierarchical cluster dendrogram, grouped by GeneSpring according to the similarity in the expression of 1115entities. Red lines represent increased mRNA level and blue lines represent decreased mRNA level, with Benjamini Hochberg False Discovery Rate corrected p-value < 5 10^−2^ and absolute fold change ≥ 2.

Hierarchical cluster analysis using the GeneSpring algorithm combined with the Euclidian similarity measure and centroid linkage rule on non-averaged status produced a combined dendrogram showing a clear distinction between the expression profiles of non-infected and infected animals (Fig 2). All microarray data can be downloaded from NCBI’s Gene Expression Omnibus (http://www.ncbi.nlm.nih.gov/geo/) with the GEO series accession number GSE70059.

It must be noted that there was no evidence of differential response between the four animals that had to be sacrificed before the end of the experiment and the two animals that survived until the end, although it was not possible to perform a statistical analysis with such a small number of animals.

### Differential gene expression analysis identifies affected immune bio-functions in *Mmm*-infected animals

**Fig 3:**
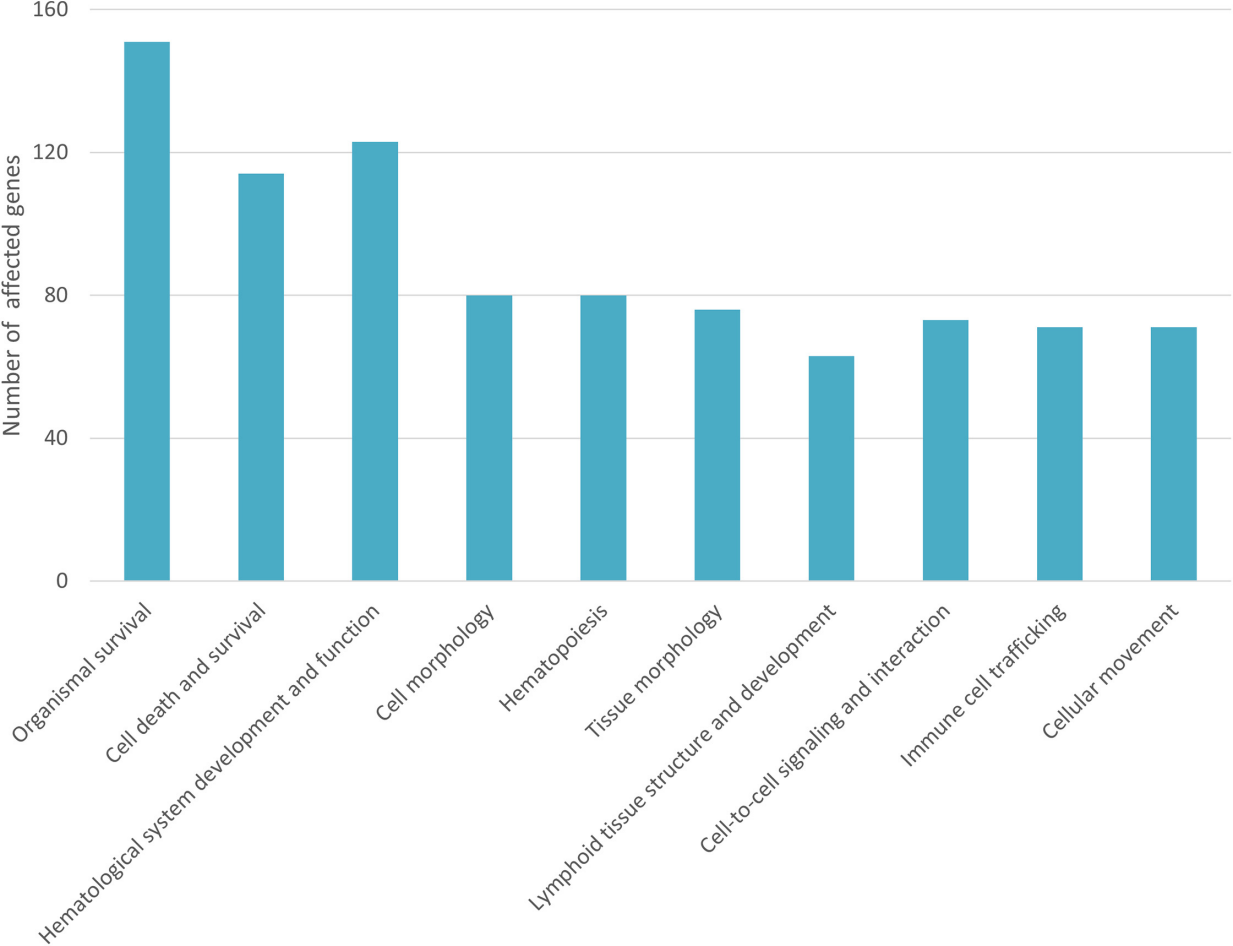
Top functions related to immune response differentially expressed in whole blood of *Mmm*-infected cattle. The ten most modulated top functions related to immune response were categorized by Ingenuity Pathway Analysis software according to p-values and z-scores. The number of differentially expressed genes in each top function is given.

To better understand the biological significance of the modulated genes, we performed bio-function analysis using IPA software. The 1,115 previously identified entities were separated into 786 mapped entities and 329 unmapped entities. A deduplication exercise on mapped entities resulted in a final list of 680 identified genes (S1 Table) of which 544 genes (i.e. 80%) were down-regulated in infected cattle. The 680 differentially-expressed genes were categorized and subcategorized according to their p-values and z-scores, revealing 10 most modulated top functions related to immune response in the infected group (Fig 3). These 10 top functions were subcategorized into bio-functions by IPA (Table 2).

**Table 2:** IPA top functions and corresponding bio-functions up- or down-regulated by *Mmm* infection.

Top functions, bio-functions and modulated genes	p-Value	z-score	genes	predicted
***Organismal survival***				
**Organismal death** ABCC1, ACTG1, ACVR1, ACVR2A, ADCYAP1R1, ADM, AFF4, AGTR1, AHI1, ANGPT1, ANP32E, APAF1, APH1A, APLP2, ARNT, ATP6AP1, B4GALT1, BAG6, BARD1, BIRC6, BUB1B, CASP8, CAST, CBL, CD28, CHRNA7, Cmah, CNGB1, CNP, CR2, CREBBP, CXCR2, DAXX, DBT, DLG1, DMTF1, DNMT3A, DYRK1A, E2F4, EHD1, EHHADH, EIF2AK3, ERCC8, F5, FUT8, FYN, G2E3, GATA6, GNAO1, GUCY1B3, HDAC8, HTR2B, HUWE1, ICOS, IFT57, IKZF1, IKZF2, IL1B, IL1R1, IL1RL1, IL1RN, ILK, INSR, IRF2, IVNS1ABP, KCNAB2, KDM6A, KIF1B, KRT19, LAMA4, LAT2, LATS1, LIN9, LRRK2, LYN, MAP3K4, MAP3K7, MCM3AP, MDM4, MED23, MED24, MEF2A, MIB1, MLL, MSH3, MTMR2, MTTP, MYCBP2, MYD88, NAB2, NBN, NCOA1, NEDD4, NFAT5, NFE2L1, PARP1, PCDHGC5, PDE4B, PDHA1, PHF14, PIP5K1C, PLAU, PLCL1, PLOD3, POR, PTAFR, PTPN11, PTPN6, PTPRC, RAC2, RAD50, RAD54B, RBL1, RBP2, RUNX2, SERPINC1, SF3B1, SHOC2, SIRT7, SKI, SLC20A1, SMAD5, SNX13, SOX5, SPEN, SPTBN1, SQSTM1, SREBF2, STAT4, STIM2, SYNE1, SYNE2, SYNJ1, TBK1, TERF2, TGM2, TOP2B, TOPBP1, UBA6, UBE3A, UBR2, USF1, USP14, VAV3, VCL, VHL, VPRBP, WASL, XRCC5, ZFPM2, ZFR	2.46.10^−3^	6.30	151	UP
***Cell death and survival***				
**Cell viability of fibroblasts** ABCC1, E2F4, EIF2AK3, FANCC, INSR, NBN, PARP1, PTPN11, XRCC5	6.8.10^−3^	-2.433	9	DOWN
**Cell survival** ABCC1, ABCC6, ABCG2, ACVR2A, ALKBH8, ANGPT1, ANLN, APAF1, ASCC3, ATG3, ATRX, BARD1, BIRC6, BUB1B, CA2, CASP8, CBL, CD28, CREBBP, DAXX, DCK, DPP3, DPP8, DST, DUSP6, E2F4, EEF2, EFNA1, EIF2AK3, FANCC, FYN, GALNT3, GATA6, HERPUD1, HSPBAP1, ICOS, IKZF2, IL1B, IL1RN, IL7R, ILK, INSR, KIF1B, KRT19, LRRK2, LYN, MALT1, MAP3K4, MAP3K7, MSH3, MYD88, NBN, NFAT5, NTN4, OGT, PARP1, PCDHGC5, PDHA1, PLAU, PLSCR1, POR, PPEF2, PPP1R8, PPP2R3A, PTAFR, PTPN11, PTPN6, PTPRC, PTPRO, RAC2, RAD50, RBBP4, SHPRH, SQSTM1, SREBF2, SSPN, STAT4, TAF4B, TBK1, TGM2, THPO, TOPBP1, USP14, VCL, VHL, XRCC5	1.89.10^−2^	-2.134	86	DOWN
***Hematological system development and function***				
**Quantity of hematopoietic progenitor cells** CASP8, CBL, CD28, CHRNA7, CREBBP, DCK, DMTF1, E2F4, EPB42, FANCC, FYN, GRAP2, HIVEP2, IKZF1, IL1RL1, IL7R, LAMA4, LYN, MLL, MYD88, NBN, PTPN11, PTPN6, PTPRC, RAC2, RASGRP1, RUNX2, SLC20A1, SMAD5, STAT4, THEMIS, THPO, TXK, VAV3, VHL, WASL, XRCC5	1.16.10^−2^	-3.431	37	DOWN
**Quantity of leukocytes** ABCG2, B4GALT1, CASP8, CBL, CD28, CHRNA7, Cmah, CNR2, CR2, CREBBP, CTSS, CXCR2, CXCR5, DCK, DMTF1, E2F4, ELMO1, FANCC, FYN, GCNT1, GRAP2, HIVEP2, ICOS, IKZF1, IL10RA, IL1B, IL1R1, IL1RL1, IL1RN, IL7R, IRF2, LYN, MALT1, MAP3K7, MDM4, MLL, MYD88, MYO9B, NBN, NFAT5, PDE4B, PLAU, PLSCR1, PTPN11, PTPN6, PTPRC, RAC2, RASGRP1, RUNX2, SIRT7, SLA, SLC20A1, STAT4, TBK1, TGM2, THEMIS, THPO, TNFRSF21, TXK, VAV3, WASL, XRCC5	3.17.10–2	-3.051	62	DOWN
**Quantity of lymphocytes** ABCG2, B4GALT1, CASP8, CBL, CD28, CHRNA7, Cmah, CNR2, CR2, CREBBP, CTSS, CXCR2, CXCR5, DCK, DMTF1, E2F4, FANCC, FYN, GCNT1, GRAP2, HIVEP2, ICOS, IKZF1, IL10RA, IL1R1, IL1RL1, IL7R, IRF2, LYN, MALT1, MAP3K7, MYD88, NBN, NFAT5, PLAU, PTPN6, PTPRC, RAC2, RASGRP1, RUNX2, SLA, STAT4, TBK1, THEMIS, TNFRSF21, TXK, VAV3, WASL, XRCC5	1.82.10–2	-2.787	49	DOWN
**Quantity of B lymphocytes** CD28, Cmah, CNR2, CR2, CREBBP, CXCR2, CXCR5, DCK, FYN, ICOS, IKZF1, IL1RL1, IL7R, IRF2, LYN, MALT1, MAP3K7, NBN, PTPN6, PTPRC, RAC2, RASGRP1, RUNX2, SLA, VAV3, WASL	1.22.10–2	-2.428	26	DOWN
**Function of lymphocytes** CBL, CD28, Cmah, CNR2, CR2, CTSS, CXCR2, DLG1, DMTF1, FYN, ICOS, IL10RA, IL16, IL1B, IL1RL1, LAT2, LYN, MAP3K4, MYD88, PANX1, PTPRC, RAD50, RASGRP1, STAT4, TBK1, TGM2, TNFRSF21, TXK	1.19.10–3	-2.408	28	DOWN
**Function of T lymphocytes** CBL, CD28, Cmah, CTSS, CXCR2, DLG1, DMTF1, FYN, ICOS, IL10RA, IL16, IL1RL1, LAT2, MAP3K4, MYD88, PANX1, PTPRC, RAD50, RASGRP1, STAT4, TBK1, TGM2, TNFRSF21, TXK	8.37.10–4	-2.395	24	DOWN
**Quantity of myeloid progenitor cells** CASP8, CREBBP, FANCC, IL1RL1, MLL, SLC20A1, THPO	5.27.10–3	-2.213	7	DOWN
**Quantity of erythroid progenitor cells** CHRNA7, CREBBP, DCK, E2F4, EPB42, IKZF1, IL1RL1, LAMA4, LYN, PTPN6, RAC2, SLC20A1, SMAD5, THPO, VHL	1.45.10–2	-2.128*	15	DOWN
**Homing of lymphocytes** ANGPT1, B4GALT1, CD28, CNR2, CXCR2, CXCR5, FLOT1, FYN, GCNT1, GNAO1, HEBP1, IL16, IL1B, IL1R1, ITGA1, LRRK2, LYN, MYD88, MYO9B, PDE4B, PIP5K1C, PLAU, PTPN6, PTPRC, PTPRO, RAC2, SERPINC1, WASL	6.57.10–3	2.995*	28	UP
**Chemotaxis of myeloid cells** ANGPT1, B4GALT1, CD28, CXCR2, FLOT1, HEBP1, IL1B, IL1R1, ITGA1, LRRK2, LYN, MYD88, MYO9B, PDE4B, PIP5K1C, PLAU, PTPN6, PTPRC, PTPRO, RAC2, SERPINC1	3.94.10–2	2.812*	21	UP
**Activation of NK cells** BLOC1S6, CD28, IL1B, IL1RL1, IL1RN, KLRF1, MALT1, MYD88, STAT4	3.08.10–2	2.39*	9	UP
**Chemotaxis of phagocytes** ANGPT1, B4GALT1, CD28, CXCR2, FLOT1, FYN, HEBP1, IL16, IL1B, IL1R1, ITGA1, LRRK2, LYN, MYD88, MYO9B, PDE4B, PIP5K1C, PLAU, PTPN6, PTPRO, RAC2, SERPINC1	2.61.10–2	3.015*	22	UP
**Recruitment of antigen presenting cells** B4GALT1, CNR2, CXCR2, FUT8, IL1B, IL1RN, LYN, MYD88	2.88.10–2	2.734*	8	UP
***Cell morphology***				
**Morphology of lymphocytes** CD28, CD46, CR2, CREBBP, DCK, FANCC, FYN, GRAP2, HIVEP2, IKZF1, IL1RN, IL7R, LYN, MALT1, PTPRC, SLA, THEMIS, XRCC5	1.57.10–4	2.37	18	UP
**Morphology of blood cells** B4GALT1, CD28, CD46, CR2, CREBBP, CXCR5, DCK, E2F4, EPB42, FANCC, FYN, GCNT1, GRAP2, HIVEP2, IKZF1, IL1RN, IL7R, LYN, MALT1, MLL, PTPRC, RAD54B, RBL1, S1PR4, SLA, THEMIS, THPO, XRCC5	5.98.10–3	2.154	28	UP
**Morphology of leukocytes** CD28, CD46, CR2, CREBBP, CXCR5, DCK, E2F4, FANCC, FYN, GCNT1, GRAP2, HIVEP2, IKZF1, IL1RN, IL7R, LYN, MALT1, PTPRC, RBL1, SLA, THEMIS, XRCC5	2.29.10–2	2.154	22	UP
***Hematopoiesis***				
**Quantity of hematopoietic progenitor cells** CASP8, CBL, CD28, CHRNA7, CREBBP, DCK, DMTF1, E2F4, EPB42, FANCC, FYN, GRAP2, HIVEP2, IKZF1, IL1RL1, IL7R, LAMA4, LYN, MLL, MYD88, NBN, PTPN11, PTPN6, PTPRC, RAC2, RASGRP1, RUNX2, SLC20A1, SMAD5, STAT4, THEMIS, THPO, TXK, VAV3, VHL, WASL, XRCC5	1.16.10^−2^	-3.431	37	DOWN
**Quantity of myeloid progenitor cells** CASP8, CREBBP, FANCC, IL1RL1, MLL, SLC20A1, THPO	5.27.10^−3^	-2.213	7	DOWN
**Quantity of erythroid progenitor cells** CHRNA7, CREBBP, DCK, E2F4, EPB42, IKZF1, IL1RL1, LAMA4, LYN, PTPN6, RAC2, SLC20A1, SMAD5, THPO, VHL	1.45.10^−2^	-2.128*	15	DOWN
***Tissue morphology***				
**Quantity of hematopoietic progenitor cells** CASP8, CBL, CD28, CHRNA7, CREBBP, DCK, DMTF1, E2F4, EPB42, FANCC, FYN, GRAP2, HIVEP2, IKZF1, IL1RL1, IL7R, LAMA4, LYN, MLL, MYD88, NBN, PTPN11, PTPN6, PTPRC, RAC2, RASGRP1, RUNX2, SLC20A1, SMAD5, STAT4, THEMIS, THPO, TXK, VAV3, VHL, WASL, XRCC5	1.16.10^−2^	-3.431	37	DOWN
**Quantity of leukocytes** ABCG2, B4GALT1, CASP8, CBL, CD28, CHRNA7, Cmah, CNR2, CR2, CREBBP, CTSS, CXCR2, CXCR5, DCK, DMTF1, E2F4, ELMO1, FANCC, FYN, GCNT1, GRAP2, HIVEP2, ICOS, IKZF1, IL10RA, IL1B, IL1R1, IL1RL1, IL1RN, IL7R, IRF2, LYN, MALT1, MAP3K7, MDM4, MLL, MYD88, MYO9B, NBN, NFAT5, PDE4B, PLAU, PLSCR1, PTPN11, PTPN6, PTPRC, RAC2, RASGRP1, RUNX2, SIRT7, SLA, SLC20A1, STAT4, TBK1, TGM2, THEMIS, THPO, TNFRSF21, TXK, VAV3, WASL, XRCC5	3.17.10^−2^	-3.051	62	DOWN
***Lymphoid tissue structure and development***				
**Quantity of lymphatic system cells** ABCG2, CASP8, Cmah, CNR2, CR2, CREBBP, FANCC, ICOS, IKZF1, IL1RL1, IL7R, ITGA1, LYN, MAP3K7, MDM4, MLL, MYD88, RBL1, SLC20A1, THPO, VHL, WASL	2.92.10^−2^	-2.575	22	DOWN
**Quantity of myeloid progenitor cells** CASP8, CREBBP, FANCC, IL1RL1, MLL, SLC20A1, THPO	5.27.10^−3^	-2.213	7	DOWN
***Cell to cell signaling and interactions***				
**Recruitment of antigen presenting cells** B4GALT1, CNR2, CXCR2, FUT8, IL1B, IL1RN, LYN, MYD88	2.88.10^−2^	2.734*	8	UP
**Activation of NK cells** BLOC1S6, CD28, IL1B, IL1RL1, IL1RN, KLRF1, MALT1, MYD88, STAT4	3.08.10^−2^	2.39*	9	UP
***Immune cell trafficking***				
**Homing of leukocytes** ANGPT1, B4GALT1, CD28, CNR2, CXCR2, CXCR5, FLOT1, FYN, GCNT1, GNAO1, HEBP1, IL16, IL1B, IL1R1, ITGA1, LRRK2, LYN, MYD88, MYO9B, PDE4B, PIP5K1C, PLAU, PTPN6, PTPRC, PTPRO, RAC2, SERPINC1, WASL	6.57.10^−3^	2.995*	28	UP
**Chemotaxis of myeloid cells** ANGPT1, B4GALT1, CD28, CXCR2, FLOT1, HEBP1, IL1B, IL1R1, ITGA1, LRRK2, LYN, MYD88, MYO9B, PDE4B, PIP5K1C, PLAU, PTPN6, PTPRC, PTPRO, RAC2, SERPINC1	3.94.10^−2^	2.812*	21	UP
**Activation of NK cells** BLOC1S6, CD28, IL1B, IL1RL1, IL1RN, KLRF1, MALT1, MYD88, STAT4	3.08.10^−2^	2.39*	9	UP
**Chemotaxis of phagocytes** ANGPT1, B4GALT1, CD28, CXCR2, FLOT1, FYN, HEBP1, IL16, IL1B, IL1R1, ITGA1, LRRK2, LYN, MYD88, MYO9B, PDE4B, PIP5K1C, PLAU, PTPN6, PTPRO, RAC2, SERPINC1	2.61.10^−2^	3.015*	22	UP
**Recruitment of antigen presenting cells** B4GALT1, CNR2, CXCR2, FUT8, IL1B, IL1RN, LYN, MYD88	2.88.10^−2^	2.734*	8	UP
***Cellular movement***				
**Recruitment of antigen presenting cells** B4GALT1, CNR2, CXCR2, FUT8, IL1B, IL1RN, LYN, MYD88	2.88.10^−2^	2.734*	8	UP
**Chemotaxis of phagocytes** ANGPT1, B4GALT1, CD28, CXCR2, FLOT1, FYN, HEBP1, IL16, IL1B, IL1R1, ITGA1, LRRK2, LYN, MYD88, MYO9B, PDE4B, PIP5K1C, PLAU, PTPN6, PTPRO, RAC2, SERPINC1	2.61.10^−2^	3.015*	22	UP
**Chemotaxis of myeloid cells** ANGPT1, B4GALT1, CD28, CXCR2, FLOT1, HEBP1, IL1B, IL1R1, ITGA1, LRRK2, LYN, MYD88, MYO9B, PDE4B, PIP5K1C, PLAU, PTPN6, PTPRC, PTPRO, RAC2, SERPINC1	3.94.10^−2^	2.812*	21	UP
**Homing of leukocytes** ANGPT1, B4GALT1, CD28, CNR2, CXCR2, CXCR5, FLOT1, FYN, GCNT1, GNAO1, HEBP1, IL16, IL1B, IL1R1, ITGA1, LRRK2, LYN, MYD88, MYO9B, PDE4B, PIP5K1C, PLAU, PTPN6, PTPRC, PTPRO, RAC2, SERPINC1, WASL	6.57.10^−3^	2.995*	28	UP
**Homing** ABCC1, AGTR1, ANGPT1, B4GALT1, CBL, CD28, CNR2, CXCR2, CXCR5, DUSP6, EFNA1, FLOT1, FYN, GCNT1, GNAO1, HEBP1, IL16, IL1B, IL1R1, ILK, ITGA1, LRRK2, LYN, MYD88, MYO9B, NEDD4, PDE4B, PIP5K1C, PLAU, PTAFR, PTPN11, PTPN6, PTPRC, PTPRO, RAC2, SEMA3B, SERPINC1, USP14, WASL	2.57.10^−2^	2.106*	39	UP

p-values were calculated with the Fisher exact test. Z-scores were calculated with the IPA z-score algorithm. The z-score predicts the direction of change of a function. An absolute z-score ≥ 2 was considered to be significant. A bias-corrected z-score* was calculated by IPA to correct dataset bias, i.e., when there are more up- than down-regulated genes or vice-versa. “genes” indicates the number of genes that are associated with each function. A function is predicted to be decreased (DOWN) if the z-score (or the bias corrected z-score) ≤ 2 and increased (UP) if the z-score≥2.

Based on the number of regulated genes, the most affected top function was *organismal survival*, with up-regulation of the *organismal death* bio-function. A total of 151 genes corresponding to this bio-function were modulated, with a p-value of 4.2 10^−3^ and a z-score of 6.3, representing a significant increase in this category. Another affected death-related top function was *cell death and survival*, with down-regulation of the *cell survival* bio-function, based on 86 modulated genes (p-value: 0.0189; z-score: -2.134).

As shown in Table 2, top functions related to *cellular development*, *morphology and functions* were affected, especially in terms of decreasing immune cell quantity: *hematopoietic progenitor cell*, *erythroid progenitors*, *myeloid progenitors*, *lymphatic system cells and B-lymphocytes*. *Function of lymphocytes and T-lymphocytes* bio-functions were also decreased. Other related immune top functions were increased by *Mmm* infection, including *immune cell trafficking*, *cellular movements* and *cell-to cell signaling and interactions*, with an increase in related bio-functions including *chemotaxis of myeloid cells*, *chemotaxis of phagocytes*, *homing of leukocytes and lymphocytes*, *recruitment of antigen presenting cells* and *activation of NK cells*.

It is however important to note that inter-animal variability was observed for some differentially-expressed genes, either in terms of magnitude or sometimes even in the direction of the changes.

### Quantitative PCR results correlate with microarray results

Validation of microarray results was performed using the Qiagen Cow RT² qPCR assay on a set of six genes involved in immune bio-functions: four up-regulated genes (IL-1RN, IL-10RA, MyD88, CXCR2) and two down-regulated genes (CD28 and SMAD-5). Single specific melt curves and no amplification in no-template controls confirmed the specificity of the RT-qPCR assays.

The RT-qPCR results were strongly correlated with microarray results for these genes, with a Pearson’s correlation coefficient of 0.973 (p-value = 0.00104). FC calculations, normalized using GAPDH and ACTB as reference genes, confirmed that CD28 and SMAD-5 gene expression was down-regulated (p-value < 0.05), whereas the other four genes were up-regulated (though with a p-value > 0.05).

Four additional genes specific for inflammation (TNFα, TLR2, IL-12B and IL-6), that were not shown to be regulated by microarray analysis, were tested by RT-qPCR. Results confirmed that they were not significantly regulated, except for IL-6, which was down-regulated, with a FC = -2.32 (p-value = 0.007) (Table 1).

## Discussion

In the current study, we compared the gene expression profiles of whole blood sampled from cattle before and during infection, at the beginning of clinical signs of acute CBPP.

### Tempus technology is a relevant and practical approach for whole blood transcriptome analyses in cattle

Several human studies have shown that, during pulmonary infection, local interaction between the infectious agent or its products and infiltrated immune cells is followed by changes in the gene expression profile of these cells, which re-circulate in whole blood [21] and express a signature of their exposure to the pathogen [22]. Global changes in gene expression in blood are a reflection of the immune response developed by the host when encountering the pathogen [23]. We decided to use this approach to explore the dynamic characterization of host-pathogen interactions in CBPP, starting by characterizing the bovine response to *Mmm* infection during acute disease, at the first appearance of clinical signs.

Peripheral whole blood is easy to collect for differential gene expression studies but it is well established that sample collection and purification greatly influence RNA quality and transcript stability [24,25]. Sheridan *et al*. [26] showed that a delay between collecting whole blood from cattle and extracting and processing of RNA could significantly alter gene expression profiles and that the magnitude of the fold-change was consistent with the length of the delay [27]. They recommended the use of Tempus^TM^ collection tubes for the stabilization of mRNA transcripts. The use of whole blood collected in RNA stabilizing reagents is currently recognized as the most reproducible method with the least variability between individuals and the minimum bias in gene expression in leukocyte cellular functions [28]. In our study, use of Tempus^TM^ technology resulted in a satisfactory quantity and quality of RNA, although samples from healthy animals had lower RIN values than those from infected animals (6.9 ± 0.4 versus 8.5 ± 0.1). This may be due to a longer interval between sampling and processing (approximately 20 weeks longer than for samples from infected animals). In conclusion, Tempus^TM^ technology proved to be a very practical way of stabilizing RNA in the field, while providing remarkable results in terms of quantity and quality of RNA for transcriptional profiling.

### Whole blood transcriptome analysis of Mmm-infected cattle reveals immunological disorders

Analysis of gene expression profiles in blood during acute CBPP revealed 680 regulated genes, with many more down-regulated genes (544, i.e. 80%) than up-regulated genes (136, i.e. 20%). It is important to note that previous *ex vivo* systemic cellular immune response studies showed neither variations in cell numeration nor significant changes in cell subsets during the disease [10], indicating that this major down-regulation was not a bias due to a drop in the number of total leukocytes or to modification of their relative abundance in the blood of infected cattle. Several studies on bovine tuberculosis also reported more down- than up-regulated genes ([29] [30], [31]). Even if the pathogenic mechanisms of *Mycobacterium tuberculosis* are probably different, the overwhelming majority of down-regulated genes in this study suggests pathogen-induced immunosuppression, which is consistent with our previous findings. Indeed, *in vitro* immunological studies showed that acute infection was characterized by a low *Mmm-*specific immune response, with low CD4^+^ T-cell recall responses and low or absent IFNγ production, in direct opposition to what was observed in chronic disease and recovered animals. [10,11]. Our results confirm that immunosuppression is a characteristic of the acute form of CBPP.

In the present study, gene modulation analysis identified an increase in the *organismal death* bio-function, with 151 regulated molecules and a decrease in *cell survival* bio-function, with 86 affected molecules (Table 2). These results do not only reflect the severe clinical signs observed in these animals during acute disease, but are also consistent with the results of previous *in vitro* analyses, showing that viable *Mmm* caused programed cell death of leukocytes [8]. This previous study also revealed that the observed effects were induced by *Mmm*-secreted components, suggesting that *Mmm* has developed mechanisms to modulate the host immune response based on secreted factors, as reviewed by Pilo *et al*. [32]. Although mycoplasmemia during *Mmm* infection is only transitory, this free exopolysaccharide can be detected in blood throughout the course of the clinical disease [3] and has been shown to play a role in *Mmm* persistence and dissemination by increasing resistance to active oxygen species secreted either by *Mmm* itself or by immune cells [7].

Other highlighted affected top functions concerned *cell morphology*, *hematological system development and hematopoiesis*, *lymphoid tissue structure and development and tissue morphology*, which were generally reduced in *Mmm*-infected animals. Some genes modulated in the present study are components of immune response pathways, or are involved in the regulation of these pathways and belong to the “common host-response to pathogens” described by Jenner and Young [33]. Indeed, reviewing numerous host-pathogen interaction microarray studies, the authors showed that a common host response exists, with common functional groups of modulated genes, representative of a general alarm signal for infection [33]. This modulated gene set has been shown to be reprogrammed by each specific pathogen and Ramilo *et al*. suggest that the final global signature in the host caused by different etiologic agents is specific [22]. Despite similarities between immune responses against pathogens, Boldrick *et al*. suggest that bacteria induce stereotyped and specific gene expression modulation [34] and Jenner and Young suggest that there is a preferential induction of genes in specific cell types and specific reprogramming of host cells by pathogens [33]. Therefore, the modulation of many of these genes involved in immune bio-functions does not represent a specific response to *Mmm* infection, since they form part of the “common host response” to pathogens, though at least part of this response may be reprogrammed by *Mmm* in a specific manner.

Finally, we identified a third group of up-regulated immune top functions, grouping *cellular movement*, *cell to cell signaling and immune cell trafficking*. These functions included *recruitment of antigen presenting cells*, *chemotaxis of phagocytes and myeloid cells*, *homing of leukocytes and activation of NK-cells*. We hypothesize that these activated NK-cells could be involved in the production of INFɣ detected in the plasma of *Mmm*-infected animals [35].

Several particular genes that were significantly modulated in our study are key components of the immune response pathways. MyD88, a gene that plays a central role in the innate and adaptive immune response (common host response), was up-regulated (FC 3.202; p-value 0.048) in infected cattle. The protein encoded by MyD88 functions as a signal transducer in IL-1 and TLR pathways, leading to NF-KB activation, cytokine secretion and inflammation. This is in agreement with the fact that acute CBPP is characterized by significant pulmonary inflammation, which causes severe clinical signs and extensive lesions. On the other hand, CD28, a gene involved in several of the modulated immune functions we identified, was down-regulated in our study (FC -2.72; p-value 0.0198). CD28 is a resting T-cell surface protein and binds to antigen presenting cells via CD80/CD86 molecules. In association with MHC, it enables T-cell activation and proliferation. Consequently, in the absence of signaling through CD28, an incomplete T-cell response occurs leading to tolerance or anergy [36]. Impaired signaling through CD28 is consistent with results of previous *in vitro* studies showing that pre-incubation of *Mmm* with T-cells followed by non-specific mitogenic stimulation led to a significant depression of lymphocyte responsiveness to the mitogen Concanavalin A [9,13]. Thus, modulation of CD28 expression by live *Mmm* deserves further investigation, as it may be a new more subtle mechanism than programed cell death to suppress the host immune response.

### Whole blood transcriptome analysis does not reflect local inflammation

Interestingly, genes involved in inflammation mechanisms were not shown modulated during infection, whereas pulmonary inflammation is the most characteristic trait of acute CBPP. Gene expression of pro-inflammatory cytokines such as the TNF superfamily, Interleukin 6, Interleukin 1, or Interleukin 12, as well as the TLR family genes, was not differentially modified in *Mmm*-infected animals. The RT-qPCR exploration of pro-inflammatory cytokines (TNFα, TLR2, IL-12B and IL-6) confirmed this lack of regulation, except for IL-6, which was statistically down-regulated. Specific exploration of a few modulated genes involved in inflammatory response (IL-1RN; IL-10RA; CXCR2 and MyD88) revealed contradictory responses, indicating complex modulation of inflammation in blood [37,38,39,40]. The fact that the local inflammatory response that characterizes acute CBPP was not reflected in whole blood in this study was surprising and differs from other studies in human pneumonia, where inflammatory cytokines [41] or genes related to inflammation were detected in blood as well as in lung [42]. On the contrary, as in our study, inflammation was not up-regulated in blood in human tuberculosis and sarcoidosis [43,44]. This may be due either to high dilution of inflammatory cells in the circulation, possibly due to sequestration of immune cells in inflamed lymph nodes, or to circulation of *Mmm*-secreted products with anti-inflammatory properties. The *Mmm* polysaccharide galactan, which can be detected in the blood of infected animals during clinical disease, particularly focuses our attention. Preliminary *in vitro* analysis using highly purified galactan [45] suggests a potent anti-inflammatory effect on bovine macrophages (P. Totte, personal communication). Another potential reason to explain the absence of inflammation-related transcripts in blood may be that resident lung cells, such as non-trafficking immune cells or epithelial/endothelial cells, are responsible for the local inflammation. This hypothesis is in agreement with the findings of Sacchini *et al*, who showed that TNFα was increased in blood of CBPP-infected cattle, although plasma levels did not increase dramatically in acute CBPP. The authors suggested that TNFα may be produced locally, then re-circulate [35].

### Whole blood transcriptome analysis clearly differentiates Mmm-infected from non-infected cattle

An interesting aspect of our results was that investigations of whole blood gene expression and hierarchical clustering analysis of differentially expressed entities clearly differentiated Mmm-infected animals from non-infected ones. We therefore suggest that *Mmm* produces a response in the blood of infected animals that is a signature of its exposure to the pathogen. In other diseases, including tuberculosis, gene expression analysis in whole blood provides valuable information for diagnostic or prognostic purposes. [29,30,46]. Transcriptional profiles of latent and cured tuberculosis patients were found to be different from those of patients with active tuberculosis and a gene set was identified to determine the form of the disease or to predict its severity [46]. Similarly, in our study, we identified 1,115 entities as an *Mmm* infection signature that would enable infected animals to be distinguished from healthy animals at the beginning of the clinical signs. This constitutes a global signature allowing the statistical discrimination of infected animals at a particular phase of the disease, though it was not possible to obtain a more limited set of regulated genes with such a signature value specific for CBPP at this stage.

## Conclusion

This work aimed to assess the use of whole blood transcriptome profiling to study bovine-mycoplasma interactions in order to explore the dynamic characterization of host-pathogen interactions in CBPP. For this preliminary study, it was decided to start by the characterization of the bovine response to *Mmm* infection during acute disease. Our results represent a “time-point picture” of the immune events occurring in the cattle-*Mmm* interplay at the appearance of the clinical signs. This global picture should be considered as the result, in blood, of the local pulmonary response (e.g. apoptosis) and the systemic events (e.g. potent anti-inflammatory properties of circulating galactan) occurring during acute *Mmm* infection. We showed that the expression of many genes was modulated, with an overwhelming majority of down-regulation of gene expression inducing an immunosuppression with consequences for important immune functions. Nevertheless, to understand the detailed immune events occurring during the disease, specific analyses of the different immune cell subpopulations, either *in vivo*, at the local site, or *in vitro*, are required. Furthermore, as our results represent a single time-point in the kinetics of acute CBPP, further work is needed to implement this approach throughout the course of the disease, which should enable the identification of molecular signatures associated with the different stages and forms of the disease. The availability of bio-signatures correlating to recovery and protection would be of particular interest, since they should facilitate the development and validation of novel vaccine formulations, avoiding costly and laborious experimental challenges in cattle.

## Supporting Information

S1 Table 680 genes differentially expressed in *Mmm*-infected cattle vs uninfected cattle (absolute fold change ≥ 2 and p-value < 5.10−^2^). (XLSX)
